# Dealloyed Ruthenium Film Catalysts for Hydrogen Generation from Chemical Hydrides

**DOI:** 10.3390/ma10070738

**Published:** 2017-07-02

**Authors:** Ramis B. Serin, Nazrin Abdullayeva, Mehmet Sankir

**Affiliations:** Department of Materials Science and Nanotechnology Engineering, TOBB University of Economics and Technology, Sogutozu Caddesi No. 43, Sogutozu 06560, Turkey; brky06@hotmail.com (R.B.S.); nabdullayeva@etu.edu.tr (N.A.)

**Keywords:** hydrogen generation, ruthenium, copper, cosputtering, dealloying, porous surface

## Abstract

Thin-film ruthenium (Ru) and copper (Cu) binary alloys have been prepared on a Teflon™ backing layer by cosputtering of the precious and nonprecious metals, respectively. Alloys were then selectively dealloyed by sulfuric acid as an etchant, and their hydrogen generation catalysts performances were evaluated. Sputtering time and power of Cu atoms have been varied in order to tailor the hydrogen generation performances. Similarly, dealloying time and the sulfuric acid concentration have also been altered to tune the morphologies of the resulted films. A maximum hydrogen generation rate of 35 mL min^−1^ was achieved when Cu sputtering power and time were 200 W and 60 min and while acid concentration and dealloying time were 18 M and 90 min, respectively. It has also been demonstrated that the Ru content in the alloy after dealloying gradually increased with the increasing the sputtering power of Cu. After 90 min dealloying, the Ru to Cu ratio increased to about 190 times that of bare alloy. This is the key issue for observing higher catalytic activity. Interestingly, we have also presented template-free nanoforest-like structure formation within the context of one-step alloying and dealloying used in this study. Last but not least, the long-time hydrogen generation performances of the catalysts system have also been evaluated along 3600 min. During the first 600 min, the catalytic activity was quite stable, while about 24% of the catalytic activity decayed after 3000 min, which still makes these systems available for the development of robust catalyst systems in the area of hydrogen generation.

## 1. Introduction

Hydrogen is one of the most promising future energy carriers, and presents a key component in several areas; for example, as a chemical agent in hydrogenation reactions, a primary reactant during carbon dioxide removal, and fuel for fuel cells [1,2,3]. Hydrogen generation can be achieved in many ways, some of which are coal gasification, electrolytic or photo-catalytic water splitting, biomass pyrolysis, and chemical hydride hydrolysis [1,2]. Among these methods, the hydrolysis of chemical hydrides (e.g., NaBH_4_, NaAlH_4_, LiH, LiBH_4_, LiH, LiAlH_4_, NaH, KBH_4_, etc.) are the most advantageous due to their high volumetric and gravimetric hydrogen densities, and they are quite stable in alkaline solution if one desires to achieve very high hydrogen generation rates in a controllable manner [1]. Sodium borohydride (NaBH_4_) as a solid hydrogen carrier has been well studied for its high content of hydrogen capacity, easy control of hydrogen generation rate at suitable operation temperatures, non-toxicity, and non-flammability [1,4].

The hydrolysis reaction of NaBH_4_ given in Equation (1) is an exothermic reaction.

NaBH_4_ (s) + (*x* + 2)H_2_O (l) → NaBO_2_*x*H_2_O (aq) + 4H_2_ (aq) + 217 kJ mol^−1^ (1)

where the *x* is a hydration factor.

If above hydrolysis reaction occurs without the presence of catalysts, the hydrogen generation rate is slow [5]. There are various catalyst systems in the area of hydrogen generation. These are generally based on noble metals, non-noble metals, alloys, and metal-free systems [6,7,8,9,10,11]. While inexpensive transition metal catalysts usually have moderate catalytic activity, noble metal catalysts exhibit excellent catalytic activity with high cost. Catalysts with various morphologies, such as powder form [12,13], particles on supported materials (e.g., carbon) [7,14], thin films coated on porous structures (e.g., nickel foam, honeycomb monolith, mesh) [15,16], or flat surfaces (e.g., copper substrate) [17] have been utilized during the hydrolysis reaction. Additionally, TiO_2_ and carbon nanotube and graphene have been recently studied as supporting agents [18,19,20]. The catalytic activity of the powdered systems ranges from 0.25 to 4.0 mL min^−1^g_catalyst_^−1^ [21]. However, film and foam systems have higher catalytic activity, which are close to 30 L min^−1^g_catalyst_^−1^ [6]. The highest catalytic activity is up to 150 L min^−1^g_catalyst_^−1^, and might be higher than has usually been achieved by the supported systems [6,21]. Catalysts in powder form should be carefully handled. Otherwise, it can lead several problems, such as difficulties in separation of the catalyst from the suspension after the reaction. Additionally, catalyst aggregation occurs and the control of the hydrogen generation rate cannot been carefully achieved. On the other hand, thin film catalysts have an extra degree of freedom to tailor their surface structures and morphologies. Catalyst in the form of thin films can be easily recovered after the reaction by simple washing processes. Therefore, thin-film catalysts in several respects might be superior to the powder form. On the other hand, it is crucial to note that as the size of the catalyst decreases, the activity of the catalyst increases greatly. There are tremendous numbers of efforts to prepare nanosized catalyst bearing very high surface area for hydrogen generation [1,2]. Similarly, the supported catalysts are preferable for their high catalytic surface area, which are usually provided by mimicking of the catalyst of the supporting layer. This is usually achieved by deposition methods including electro, electroless and pulsed laser deposition, dip coating, ultrasonic spray, as well as chemical reduction methods [1,22]. Again, the resulted morphology after deposition is strongly related to the substrate morphology. Therefore, the thickness is quite important after deposition. In other words, the catalyst layer should be thin enough, otherwise continuous film formation on the porous substrate occurs and this eventually causes clogging of the porous media. However, using dealloyed catalysts may solve this problem. Dealloying or selective dissolution includes the removal of one or more components out of an alloy. It is a process providing fast and direct production of nanoporous materials, which have high surface area and are of considerable interest for use in various applications such as sensors, electrodes, hydrogen storage, and catalysis [6,23,24,25]. In this study, we are reporting the one-step formation of thin-film catalyst by dealloying Ru-Cu alloys which are prepared by cosputtering of Cu and Ru at various compositions for use in the area of hydrogen generation. It is worth mentioning that Ru and Cu form peculiar alloys with a positive heat of formation. Alonso et al. have calculated the free energy of Ru and Cu alloys at various Cu atomic fractions [26]. Additionally, we also present the one-step synthesis of nanoforest-like structure formation which might be useful in other areas requiring Ru precursor as catalysts or electrodes. The influence of sputtering time and power of Cu, dealloying time, and etchant concentration during dealloying on hydrogen generation rates have also been explored.

## 2. Materials

Sodium borohydride (NaBH_4_, 98 wt %) was purchased from Sigma Aldrich (Schnelldorf, Germany). Ru-Cu binary alloys have been prepared by cosputtering technique using Vaksis Midas PVD-MT/2M2T magnetron sputtering system (Ankara, Turkey). Ru and Cu targets (purity 99.99 wt %) were provided from Plus Materials (Atlanta, GA, USA). Teflon™ was provided from Oz-Ka Metal Inc. (Los Angeles, CA, USA) and was used as a backing layer of the catalyst during cosputtering.

### Alloying, Dealloying Methods and Hydrogen Generation and Durability Tests

A Teflon™ backing layer was coated by Cu and Ru atoms simultaneously in the presence of substrate rotation in a vacuum chamber (*P* = 6.3 × 10^−3^ Torr) with argon feed rate of 45 sccm (Vaksis Midas PVD-MT/2M2T, Ankara, Turkey). Sputtering power of Ru target was kept at 50 W in all coatings. On the other hand, the sputtering power of Cu target was selected as 30, 50, 100, 200, and 300 W. The coating time was varied from 15 to 90 min.

After deposition, a dealloying process was applied for Ru-Cu alloys. Sulfuric acid (H_2_SO_4_, 96 wt %, Sigma-Aldrich) was used as etchant to dealloy Cu atoms from alloys. The dealloying process was carried out in an ultrasonic sonicator (Kudos SK3310LHC, Shanghai, China, vibration frequency = 35 kHz). Dealloying time ranged from 30 to 120 min, and the acid concentration was varied from 6 to18 M.

**Figure 1:**
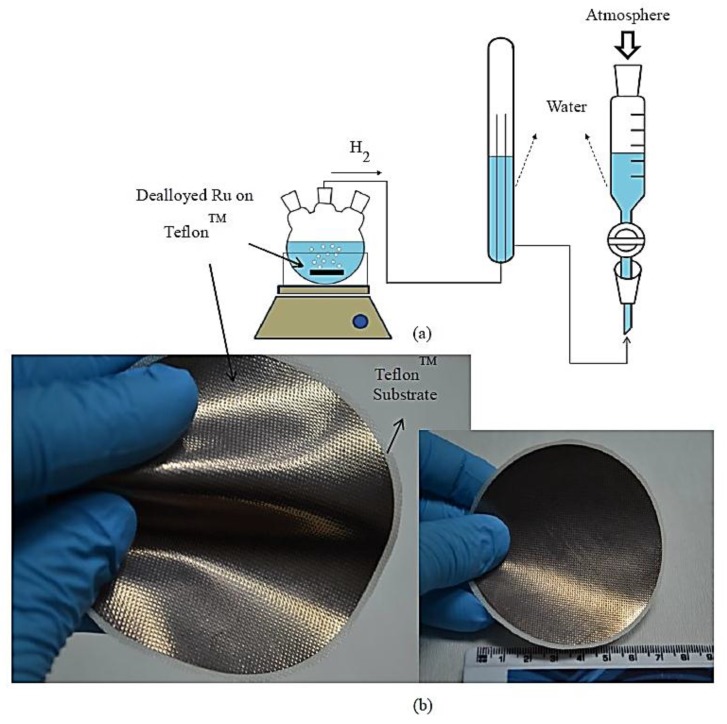
(**a**) Hydrogen generation measurement system; (**b**) Dealloyed Ru catalyst on Teflon™ substrate.

Finally, dealloyed films were used in the hydrogen generation measurement tests. As seen in Figure 1a, the hydrogen generation system included a three-neck reaction chamber (including catalyst and NaBH_4_ solution) and two cylindrical tubes. Hydrogen generation rate is reported by using water displacement technique, wherein the volume of hydrogen generated at a given interval is measured by reading the volume of the drained water in the cylindirical tube. In other words, produced hydrogen volume is measured according to the change of water level in the gas burette and recorded over time during the experiments. The volume of raised water level is equivalent to the volume of hydrogen produced. On the other hand, the durability tests include the continuing measurement of the produced hydrogen with units of mL min^−1^ in a reactor described above equipped with a mass flow meter. All of the experiments were conducted with 2.46 g NaBH_4_ in 150 mL deionized water, and the area of the thin film catalyst was about 6.65 cm^2^. The weight percent values of both Cu and Ru in alloys were detected via energy dispersive X-ray (EDX) microanalysis.

## 3. Results and Discussion

**Figure 2:**
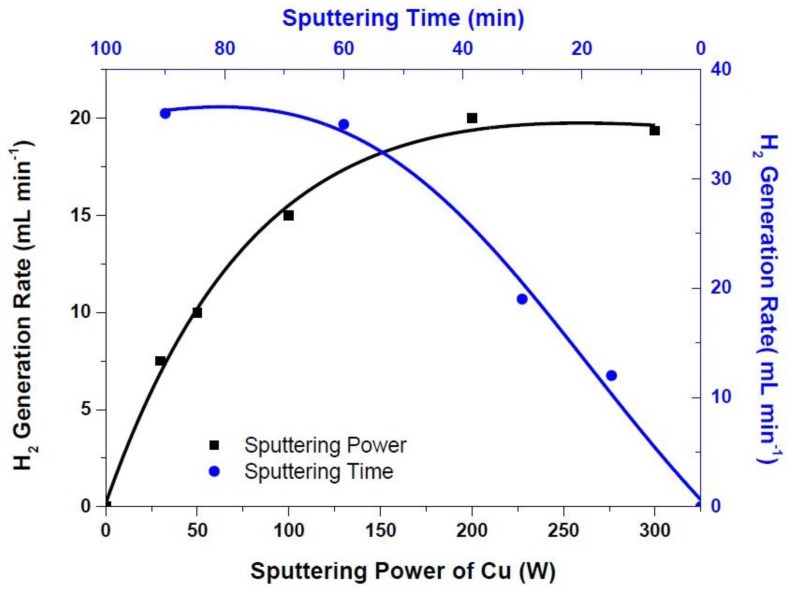
Influence of sputtering power (sputtering and dealloying time and the acid concentration are both 60 min and 15 M, respectively) and sputtering time of Cu (the sputtering powers of Ru and Cu were kept as 50 and 200 W, respectively, dealloying time was 60 min, and the acid concentration was 15 M ) on hydrogen generation rates.

Alloys from Ru and Cu were prepared by cosputtering on the Teflon™ backing layer. That is, thin film flexible Ru-Cu alloy sheets on Teflon™ backing layer that can be cut into any shape with chemical inertness during the hydrolysis reaction have been achieved (Figure 1b). Dealloying of Cu in Ru-Cu alloy sheets resulted in the formation of nanostructured precious metal, which was then utilized as hydrogen generation catalysts. Various parameters were tailored to achieve very high hydrogen generation rates. One of these parameters is the sputtering power of Cu. Therefore, it was varied between 30 and 300 W while sputtering power of Ru was kept at 50 W. As seen in Figure 2, the hydrogen generation rate increased from 8 mL min^−1^ to 20 mL min^−1^ when increasing the sputtering power of Cu from 30 to 200 W. The maximum hydrogen generation rate was measured as 20 mL min^−1^ when Cu and Ru sputtering powers were 200 and 50 W, respectively. De-alloying was achieved by using sulfuric acid in an ultrasonic sonicator. Dealloying time and the acid concentration were about 90 min and 15 M, respectively. It seems that the sputtering power of 200 W is a threshold, since the hydrogen generation rate did not change once sputtering power of Cu reached 300 W. As a result, the optimum sputtering power of Ru-Cu alloy was found to be 50 and 200 W for Ru and Cu, respectively.

We have also investigated the effect of cosputtering time during Ru-Cu alloy formation on hydrogen generation rate. The Ru-Cu alloy coating by sputtering time was varied from 15 to 90 min. On the other hand, the sputtering powers of Ru and Cu were kept as 50 and 200 W, respectively. As can be seen in Figure 2, the hydrogen generation rates increased from 12 to 35 mL min^−1^ when cosputtering time was increased from 15 to 60 min, which is another threshold indicating optimum cosputtering time. One easily concludes that as cosputtering time increases, the thicknesses of the alloys also increase. During dealloying or etching steps, thin alloys (thinner than 1 μm) can easily delaminate from the surface, and the amount of precious metal on the surface dramatically decreases. Therefore, the ideal thickness for the alloy to utilize them in the hydrogen generation tests should be about 1.5 μm, which can be easily obtained after 60 min cosputtering time.

**Figure 3:**
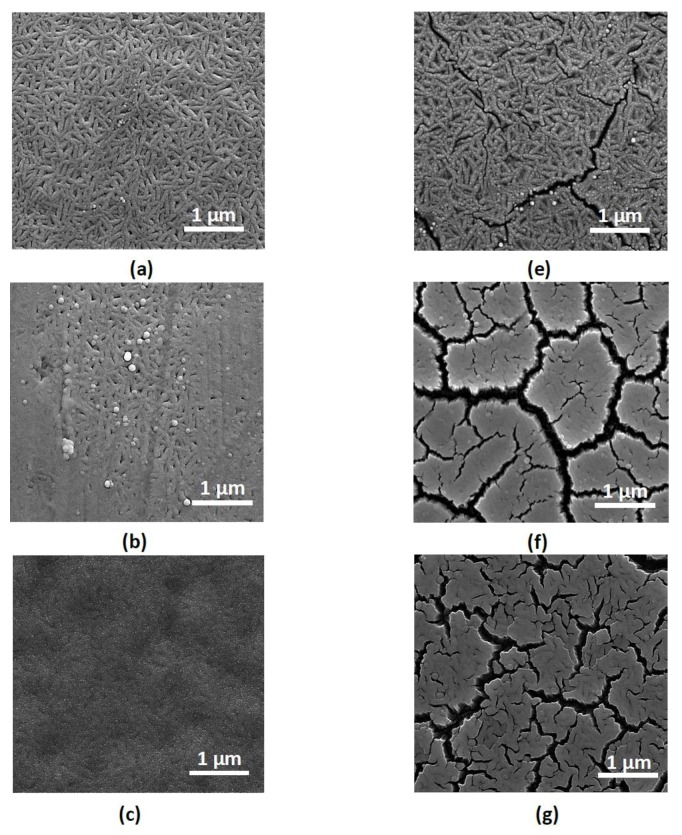

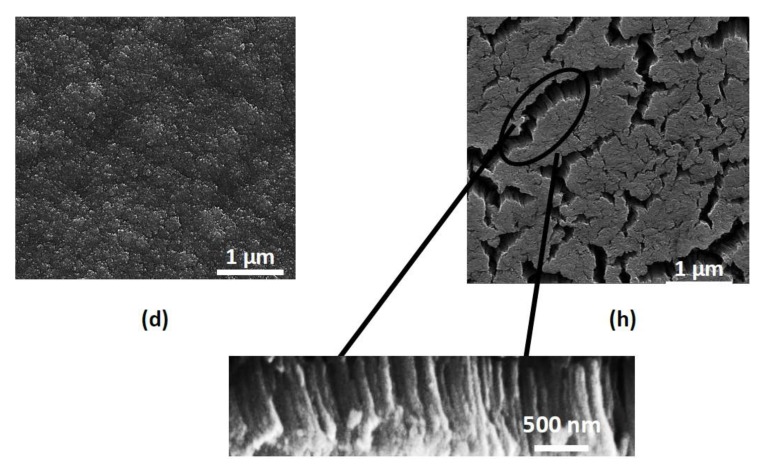
Scanning electron microscopy images of alloyed (**a**–**d**) and dealloyed (**e**–**h**) catalyst at various Cu alloying sputtering powers: (**a**) 30 W; (**b**) 50 W; (**c**) 100 W; (**d**) 200 W; (**e**) 30 W; (**f**) 50 W; (**g**) 100 W; (**h**) 200 W, where the sputtering power of Ru is kept as 50 W.

Morphological changes of thin-film catalysts better describe the influence of the sputtering power of Cu (Figure 3). It can be clearly stated that nanoporous alloys have been prepared with lower Cu sputtering power (Figure 3a,b). Although nanoporous morphologies are obtained, the catalytic activities of the dealloyed thin films are too low (lower than 10 mL min^−1^). At lower Cu sputtering power, the Ru content is also low, which is indicated from very thin film formation of catalyst (0.5 μm). When the sputtering power reaches 100 W and beyond, continuous film formation of alloys occur (Figure 3c,d). The average thickness of these dealloyed films is about 1.5 μm. Therefore, dealloying of the thicker alloys results in higher catalytic activity than that of thinner alloys. It has also been demonstrated that the homogeneous crack formation occurs for all samples after the dealloying step (Figure 3e,h). Interestingly, the crack formation shows that there are Ru nanoforest-like structures formed between the supporting layer and the surface of the catalyst layer after dealloying (Figure 3h). On the other hand, Ru nanoforest-like structures—which have previously been prepared by a template [27]—have been formed within this study by a simple one-step template-free dealloying process.

**Figure 4:**
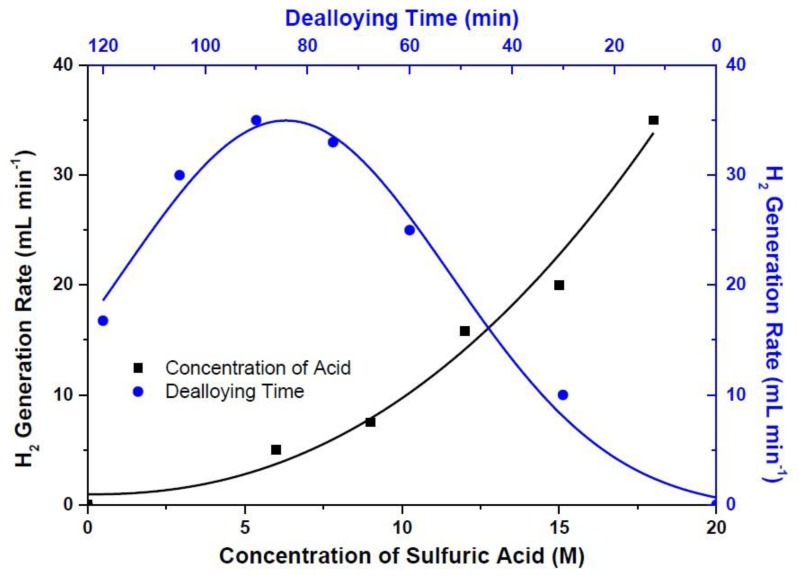
Influence of acid concentration (sputtering power of Ru and Cu and sputtering time are 50 and 200 W and 60 min, respectively, and dealloying time was 60 min) and dealloying time (sputtering power of Ru and Cu and sputtering time are 50 and 200 W and 60 min, respectively, and the acid concentration is 18 M) on hydrogen generation rates.

Effect of acid concentration in the dealloying process on hydrogen generation rate has also been studied. Hence, the hydrogen generation rate has been measured at various acid concentrations ranging from 6 to 18 M, which is the highest available concentration of the stock solution. Figure 4 demonstrates that the hydrogen generation rates scale with the acid concentration, since the hydrogen generation rates raise from 5 to 35 mL min^−1^ once the acid concentration increases from 6 to 18 M. It has been concluded that the best etchant concentration is limited to the highest available acid concentration.

Another variable is dealloying time, and has been varied in order to observe the influence of dealloying time on hydrogen generation rate. Dealloying time is ranged from 15 to 120 min while sulfuric acid concentration and sputtering powers of Ru and Cu alloy are 18 M, 50, and 200 W, respectively. As seen in Figure 4, hydrogen generation rate reached a maximum of 35 mL min^−1^ with the dealloying time of 90 min. It can be seen from the same figure that the increase in dealloying time after 90 min reduced the hydrogen generation performances. In order to better understand the effect of dealloying on the hydrogen rates, the composition of the alloys was investigated. According to EDX analysis, the bare alloy composition consists of Ru and Cu with the weight percent of 7.12 and 92.88, respectively (Table 1). The ratio of Ru to Cu of bare alloys is too low, at 0.08. Once the dealloying was achieved in just 30 minutes, the weight percent Ru increased from 7.12 to 40.75; however, it was decreased from 92.88 to 59.25 for Cu. The ratio of Ru to Cu then increased by almost nine times and reached 0.69 in 30 min dealloying. This increase in Ru and decrease in Cu contents continued and the weight percent of Ru and Cu at 90 min dealloying reached 93.84 and 6.16, respectively. This means that an approximately 190 times increase in Ru to Cu ratio was observed after 90 min dealloying. The key to increasing hydrogen generation performances with increasing dealloying time is raising the Ru to Cu ratio. This increase continued along to 120 min dealloying, and the ratio of Ru to Cu became 26.40, which corresponds to a 330-times increase compared to that of bare alloy. Since dealloying after 90 minutes caused delamination of the catalyst layer, the hydrogen generation rates suffered from this delamination. When dealloying time reached 105 min, hydrogen generation rates decreased from 35 to 30 mL min^−1^, and were further lowered to 17 mL min^−1^ at 120 min dealloying due to the excessive amount of dealloying with prolonged dealloying time.

**Figure 5:**
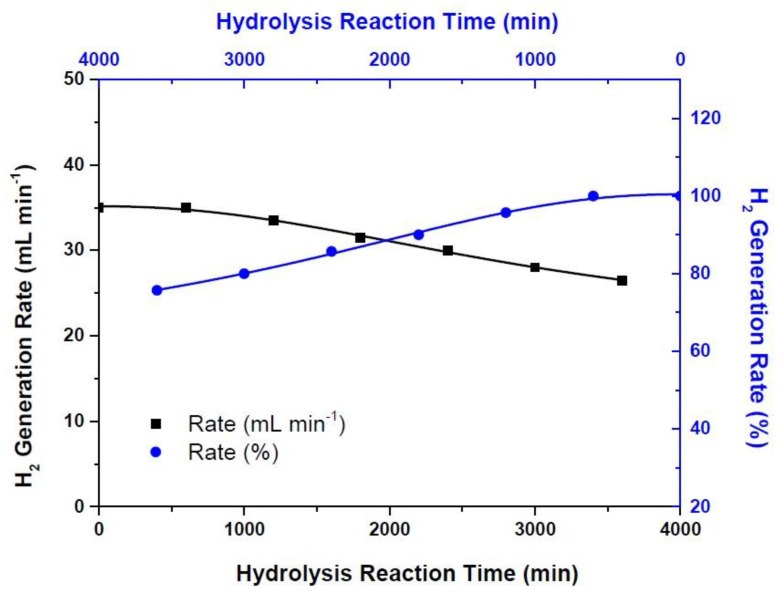
The durability tests of the hydrogen generation catalysts (the percent hydrogen generation rate has been calculated by choosing the highest hydrogen generation rate of 35 mL min^−1^ as 100%).

The durability tests give us information about the long-term performances of the catalyst system developed in this study. Therefore, the catalyst system resulted with the highest possible hydrogen generation rate where the sputtering power of Cu and sputtering time were 200 W and 60 min and the dealloying time and the concentration of the acid were 90 min and 18 M, respectively, has been tested along 3600 min continuously. As can be seen in Figure 5, hydrogen generation rate is highly stable and does not change along the first 600 min. However, hydrogen generation performance of thin-film catalyst system decreased from 35 mL min^−1^ to 26.5 mL min^−1^ at the end of the 3600 min test period. This corresponds to approximately 24% of the initial rate, in which the remaining rate is high enough to utilize this catalyst system in many applications such as proton exchange membrane (PEM)-type fuel cells requiring hydrogen gas [11].

## 4. Conclusions

In this work, binary alloys of Ru and Cu have been cosputtered on a Teflon™ backing layer. Sulfuric acid solution at various concentrations from 6 to 18 M has been used for the dealloying process. Dealloying time has also been varied, and hydrogen generation rate performances of the catalysts have been evaluated. The optimum catalyst preparation method resulting the highest possible hydrogen generation rate of 35 mL min^−1^ can be achieved in a way that the sputtering time and power of Cu are 60 min and 200 W, whereas the acid concentration and dealloying time are 18 M and 90 min, respectively. It has also been demonstrated that the template-free nanoforest-like structure formation after dealloying process has been achieved. Finally, the durability tests conclude that the hydrogen generation rates are highly stable along first 600 min while there is about 24% decay after 3000 min, which is enough for further utilization of this system in applications demanding rapid hydrogen generation.

## Figures and Tables

**Table 1:** Ru and Cu content at various dealloying times (the sputtering powers of Ru and Cu were 50 and 200 W, respectively, while the sputtering time and sulfuric acid concentration were 60 min and 18 M, respectively, and results have been obtained via energy dispersive X-ray (EDX) analysis).

Catalysts	Dealloying Time (min)	Ru (wt %)	Cu (wt %)	Ratio (Ru/Cu)
Bare Alloy	Without Dealloying	7.12	92.88	0.08
Dealloyed	30	40.75	59.25	0.69
	60	83.84	16.16	5.19
	75	92.90	7.10	13.08
	90	93.84	6.16	15.23
	105	94.14	5.86	16.06
	120	96.35	3.65	26.40
